# Neonatal mortality trends in the 21st century: findings from the Global Burden of Disease Study 2021

**DOI:** 10.1016/j.jped.2026.101519

**Published:** 2026-02-17

**Authors:** Dongqing Gu, Xiaofei Zheng, Yan Zhou, Fengjie Tan, Rui Gui, Linna Wei, Lubin Liu

**Affiliations:** a Chongqing Health Center for Women and Children (Women and Children’s Hospital of Chongqing Medical University), Department of Obstetrics and Gynecology, Chongqing, China; b Chongqing Health Center for Women and Children (Women and Children’s Hospital of Chongqing Medical University), Department of Scientific Research and Foreign Affairs, Chongqing, China; c Third Military Medical University (Army Medical University), Southwest Hospital, Department of Infectious Diseases, Chongqing, China; d Chongqing Health Center for Women and Children (Women and Children’s Hospital of Chongqing Medical University), Department of Newborn Delivery Center, Chongqing, China

**Keywords:** Neonatal mortality, Cause-specific mortality, Temporal trends, Sustainable Development Goal 3.2, Global Burden of Disease

## Abstract

**Objective:**

Neonatal mortality remains a significant global health challenge, particularly in low- and middle-income countries. This study aimed to analyze the trends and causes of neonatal mortality from 2000 to 2021.

**Methods:**

Data on all-cause and cause-specific mortality were obtained from the Global Burden of Disease Study 2021. Joinpoint regressions were employed to calculate the annual percentage change and the average annual percentage change. Pearson's correlation was utilized to estimate the relationships between mortality rates and the Human Development Index.

**Results:**

The global all-cause neonatal mortality rate has declined by an average of 2.1% per year since 2000, while the absolute number of neonatal deaths remained at 2.19 million globally in 2021. Neonatal disorders continued to be the leading cause of death among neonates in 2021, followed by congenital birth defects, lower respiratory infections, sexually transmitted infections excluding HIV, diarrheal diseases, and malaria. The neonatal mortality rate varied significantly across different countries and territories, ranging from 0.61 per 1000 livebirths in Andorra to 55.35 per 1000 livebirths in South Sudan. By 2021, 120 countries (58.8%) had achieved the Sustainable Development Goal 3.2 target. Additionally, strong negative correlations were found between mortality rates and Human Development Index (*r* = -0.867, *P* < 0.001).

**Conclusions:**

Despite significant progress, regional disparities persist; only half of the countries achieved the Sustainable Development Goal 3.2 target by 2021. These findings emphasize prioritized interventions for neonatal disorders in low/middle-income countries and highlight socioeconomic development as critical to equitable mortality reduction within public health frameworks.

## Introduction

Neonatal mortality rates serve as a key indicator of a nation's healthcare quality and socioeconomic development [1]. The World Health Organization (WHO) estimates that approximately 2.5 million neonates die each year, with many of these deaths occurring in low- and middle-income countries [2]. Since 2000, the Millennium Development Goals (MDGs) have been adopted to reduce deaths from communicable diseases such as HIV, tuberculosis, and malaria (MDG 6); maternal mortality (MDG 5); and child mortality (MDG 4). Furthermore, Sustainable Development Goal (SDG) 3.2 specifically aims to quantify the preventable portion of child mortality. It states, “By 2030, end preventable deaths of newborns and children under 5 years of age, with all countries aiming to reduce neonatal mortality to at least as low as 12‰ LB (live births) and under-5 mortality to at least as low as 25‰ LB” [3,4]. Although neonatal deaths have decreased by 44 % since 2000, they accounted for a larger proportion (47 %) of all deaths among children under-5 in 2022 [5]. Globally, the mortality rate of children under-5 decreased from 71.2‰ LB in 2000 to 37.1 in 2019; however, the mortality rate of neonates declined at a slower pace [1].

Analyzing the factors contributing to neonatal mortality, high-risk elements can be identified, leading to the development of targeted preventive measures and intervention strategies aimed at reducing the neonatal mortality rate. Numerous studies have been conducted to highlight the multifaceted nature of this public health challenge [6, 7, 8, 9, 10], and to address the leading causes of neonatal deaths, which include preterm birth, birth asphyxia, and neonatal infections [11, 12, 13]. Recent analyses indicate that, while progress has been made in reducing neonatal mortality rates, disparities persist across different regions of the world [1,8,14].

The Global Burden of Disease Study (GBD), a collaborative initiative led by the Institute for Health Metrics and Evaluation (IHME) at the University of Washington, has been instrumental in tracking health trends and disparities worldwide [15]. To address this critical gap, this study provides an in-depth analysis of neonatal mortality from 2000 to 2021 using the latest data from the GBD 2021 [16,17]. The authors examine all-cause and cause-specific mortality rates across global, regional, and national levels, analyze temporal trends, identify leading causes, and assess their correlation with socioeconomic development. The findings would provide critical insights for formulating and optimizing public health policies to reduce global neonatal mortality rates.

## Methods

### Patient and public involvement

The study used publicly available data and did not involve identified human information; therefore, no ethics approval or informed consent was needed.

### Study design and data sources

This study is an ecological trend analysis based on a time series of cross-sectional estimates. The primary data were derived from the annual cross-sectional estimates provided by the GBD 2021 study for the period 2000–2021. The authors analyzed these data to examine temporal trends at the population level. Specifically, all-cause and cause-specific mortality data for neonates (aged < 28 days) were obtained using the online Global Health Data Exchange (GHDx) query tool (https://vizhub.healthdata.org/gbd-results/). For countries with limited or absent direct data, GBD employs disMod-MR 2.1, a Bayesian meta-regression tool, and other statistical models that synthesize all available data (e.g., from surveys, censuses, verbal autopsies, and scientific literature) while incorporating relevant covariates (e.g., socioeconomic indicators, healthcare access) to generate internally consistent estimates of mortality rates and their causes [18]. A key output of this process is the 95 % uncertainty interval (UI), which quantifies the statistical uncertainty around each estimate, reflecting both data scarcity and model variability. Specifically, the 95 % UIs were defined by the 25th and 75th percentiles of the ordered 1000 estimates derived from the GBD algorithm.

Causes of neonatal mortality were classified according to the GBD hierarchical cause list, which organizes diseases and injuries into four levels of increasing specificity. Level 1 represents broad cause categories (e.g., communicable diseases), Level 2 includes intermediate subgroups (e.g., neonatal disorders), Level 3 comprises specific diseases or conditions (e.g., neonatal preterm birth), and Level 4 provides further etiological detail where applicable. In this study, the term “leading cause” refers to the disease or condition that accounted for the highest mortality rate within a given population (global, regional, or national) for the specified year. The present analysis primarily focused on identifying leading causes of death at Levels 1 through 3 across global, regional, and national levels.

The data encompassed a total of 204 countries and territories, representing comprehensive global coverage. Countries and territories were categorized into five Sociodemographic Index (SDI) groups (low, low-middle, middle, high-middle, and high SDI), which is a composite measure developed by the Institute for Health Metrics and Evaluation (IHME) that combines indicators of income per capita, educational attainment, and total fertility rate [18]. Additionally, they were grouped into 21 GBD regions based on geographical contiguity. Furthermore, neonatal deaths were stratified by age into two groups: 0–6 days (early neonatal) and 7–27 days (late neonatal).

The Human Development Index (HDI), obtained from the United Nations Development Programme (UNDP) (https://hdr.undp.org/data-center/human-development-index#/indicies/HDI), is a composite statistic that reflects key dimensions of human development, including the average achievements in leading a long and healthy life, educational attainment, and a respectable standard of living [19]. HDI values range from 0 to 1, with higher values indicating greater human development.

### Statistical analysis

A joinpoint regression analysis was conducted to evaluate the temporal trends in mortality rates from 2000 to 2021. The optimal number and location of joinpoints were determined using a permutation test [20]. Model fit was assessed by examining the residuals to ensure they were randomly distributed and that no systematic patterns were present, confirming the adequacy of the selected model. This statistical methodology is frequently utilized in epidemiological research to evaluate temporal trends in disease prevalence or mortality [20]. Specifically, the annual percentage change (APC) and average annual percentage change (AAPC) with 95 % confidence intervals (CIs) were calculated using the logarithmic linear model (ln *y* = xb) and the Empirical Quantile Method [21,22]. The APC quantifies the change per year, while the AAPC summarizes the trend over the entire study period (2000–2021). Trends were interpreted as increasing, decreasing, or stable based on whether the 95 % CI for the AAPC excluded or included zero.

Pearson's correlation was used to examine the relationships between the mortality rate and the AAPC with the HDI. All statistical analyses were performed using the Joinpoint Regression Program (version 5.0.2) and R (version 4.3.3). A two-tailed *P*-value of <0.05 was considered indicative of statistically differences.

## Results

### Global trends

**Fig. 1:**
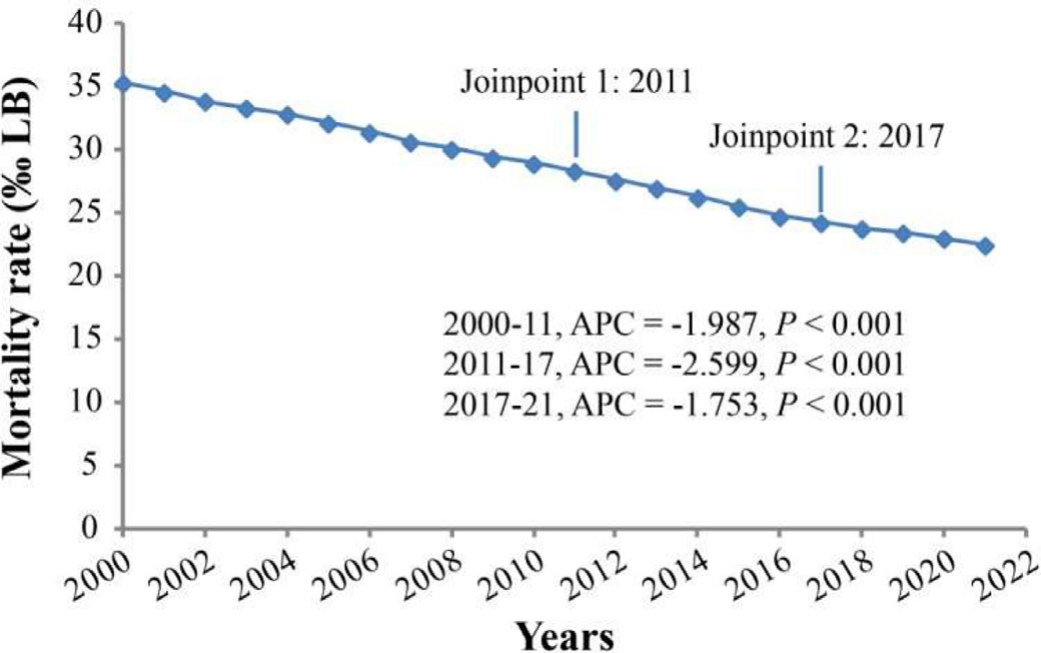
Joinpoint regression analysis of global neonatal mortality from 2000 to 2021. APC, annual percentage change; LB, live births.

The absolute number of neonatal deaths was 3.46 million (95 % UI: 3.29 to 3.66) in 2000, decreasing to 2.19 million (95 % UI: 1.90 to 2.53) in 2021. The mortality rate declined from 35.31‰ LB (95 % UI: 33.55 to 37.34) in 2000 to 22.46 (95 % UI: 19.45 to 25.99) in 2021, with an AAPC of −2.1 % (95 % CI: −2.3 to −2.0) (Table 1). Joinpoint regression analysis identified significant changes in neonatal mortality in 2011 and 2017 (Figure 1).

**Table 1:** Current status and temporal trends of neonatal mortality from 2000 to 2021.

Characteristics	In 2000		In 2021		2000–2021	
	Counts (million)	Death (‰ LB)	Counts (million)	Death (‰ LB)	AAPC (95 % CI)	*P*
All cause	3.46 (3.29 to 3.66)	35.31 (33.55 to 37.34)	2.19 (1.90 to 2.53)	22.46 (19.45 to 25.99)	−2.1 (−2.3 to −2.0)	<0.001
Cause-specific						
Communicable, maternal, neonatal, and nutritional diseases	3.10 (2.94 to 3.28)	31.61 (29.95 to 33.45)	1.94 (1.68 to 2.25)	19.95 (17.24 to 23.04)	−2.1 (−2.3 to −2.0)	<0.001
Non-communicable diseases	0.34 (0.28 to 0.41)	3.46 (2.87 to 4.23)	0.22 (0.19 to 0.28)	2.29 (1.91 to 2.84)	−1.9 (−2.0 to −1.9)	<0.001
Injuries	0.02 (0.02 to 0.03)	0.24 (0.20 to 0.28)	0.01 (0.01 to 0.02)	0.13 (0.10 to 0.17)	−3.2 (−4.0 to −2.5)	<0.001
Other COVID-19 pandemic-related outcomes	-	-	0.004 (0.002 to 0.007)	0.09 (0.05 to 0.15)	-	-
Sex						
Male	1.97 (1.86 to 2.09)	38.78 (36.53 to 41.05)	1.26 (1.07 to 1.47)	24.93 (21.31 to 29.16)	−2.1 (−2.2 to −1.9)	<0.001
Female	1.49 (1.43 to 1.56)	31.57 (30.20 to 33.05)	0.93 (0.82 to 1.06)	19.82 (17.46 to 22.59)	−2.2 (−2.3 to −2.0)	<0.001
Age						
0 - 6 days	2.58 (2.45 to 2.73)	104.19 (98.91 to 110.19)	1.74 (1.51 to 2.02)	70.99 (61.41 to 82.30)	−1.8 (−1.9 to −1.7)	<0.001
7 - 28 days	0.88 (0.84 to 0.93)	12.03 (11.45 to 12.64)	0.45 (0.39 to 0.52)	6.15 (5.35 to 7.07)	−3.1 (−3.3 to −3.0)	<0.001
SDI						
Low SDI	1.12 (1.07 to 1.17)	54.07 (51.45 to 56.61)	0.97 (0.82 to 1.15)	35.82 (30.34 to 42.32)	−1.9 (−2.0 to −1.9)	<0.001
Low-middle SDI	1.45 (1.35 to 1.55)	47.77 (44.63 to 51.22)	0.84 (0.72 to 0.98)	28.58 (24.57 to 33.55)	−2.4 (−2.5 to −2.3)	<0.001
Middle SDI	0.69 (0.65 to 0.73)	24.97 (23.46 to 26.59)	0.31 (0.27 to 0.36)	12.82 (11.05 to 14.98)	−3.1 (−3.3 to −2.9)	<0.001
High-middle SDI	0.16 (0.15 to 0.17)	14.61 (13.6 to 15.63)	0.04 (0.04 to 0.05)	4.78 (4.25 to 5.34)	−5.2 (−5.6 to −4.8)	<0.001
High SDI	0.05 (0.04 to 0.05)	5.36 (5.20 to 5.53)	0.03 (0.02 to 0.03)	3.28 (3.04 to 3.53)	−2.3 (−2.7 to −1.9)	<0.001

AAPC, average annual percent change; CI, confidence interval; COVID-19, Coronavirus Disease 2019; LB, live births; SDI, sociodemographic index.

At the broadest categorical level (Level 1), communicable, maternal, neonatal, and nutritional diseases were the primary causes of neonatal mortality, accounting for 3.1 million deaths (95 % UI: 2.94 to 3.28) in 2000 and 1.94 million deaths (95 % UI: 1.68 to 2.25) in 2021 (Table 1). Within this overarching group, neonatal disorders (Level 3) remained the leading specific cause of death among neonates in 2021, followed by congenital birth defects, lower respiratory infections, sexually transmitted infections excluding HIV, diarrheal diseases, and malaria (Fig. S1). Neonatal disorders were the leading cause of death for both the early (0–6 days) and late (7–28 days) neonatal periods, with congenital birth defects and lower respiratory infections ranking as the second leading cause in each respective group (Fig. S1). Analysis revealed a largely overlapping set of top ten causes between age groups, underscoring the persistent burden of conditions like neonatal disorders and congenital defects across the neonatal period (Fig. S1). The principal distinction was the appearance of foreign body and stroke ranked among the top ten causes only in the 0–6 day group, while malaria and sudden infant death syndrome were unique to the top ten list for the 7–28 day group.

Male newborns consistently had a higher mortality rate than females (Table 1), although the distribution of the top ten causes of death did not differ significantly by sex (Fig. S2). Neonatal deaths were concentrated in the first week of life, accounting for 70.99‰ LB (95 % UI: 61.41 to 82.30) of all neonatal mortality in 2021. Mortality rates declined significantly from 2000 to 2021 in both the early and late neonatal periods, with the most pronounced average annual decrease observed in the late neonatal period (AAPC: −3.1 %, 95 % CI: −3.3 to −3.0; Table 1).

In 2021, regions with low-SDI had the highest mortality rate at 35.82‰ LB (95 % UI: 30.34 to 42.32), while regions with high SDI reported the lowest mortality rate at 3.28‰ LB (95 % UI: 3.04 to 3.53) (Table 1). Throughout the study period, all SDI regions demonstrated a varying decline in neonatal mortality, with the most significant decrease observed in regions classified as high-middle SDI, which had an AAPC of −5.2 % (95 % CI: −5.6 to −4.8) (Table 1). Notably, there were significant reductions in mortality due to communicable, maternal, neonatal, and nutritional diseases, as well as non-communicable diseases and injuries among both male and female neonates in regions with low SDI, low-middle SDI, middle SDI, and high-middle SDI (Fig. S3).

### Regional trends

**Fig. 2:**
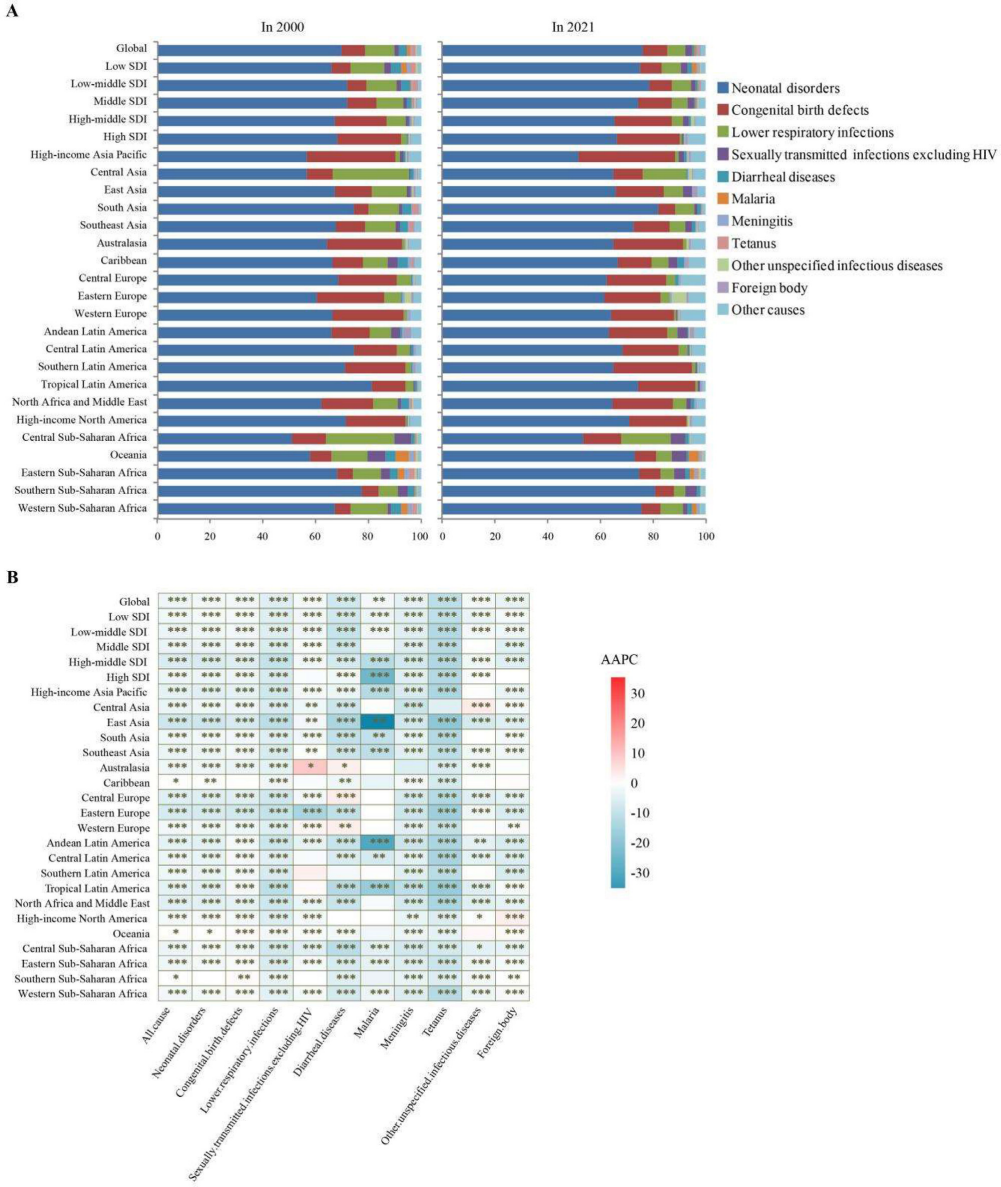
The leading 10 level 3 causes of neonatal mortality by region and SDI. A. The proportion of cause-specific neonatal mortality, B. the AAPC of the cause-specific neonatal mortality rate. *P*-values were calculated using Joinpoint regression analysis. Significance levels are indicated as * < 0.05, ** < 0.01, *** < 0.001. SDI, Socio-demographic Index; AAPC, average annual percentage change.

In 2021, Western Sub-Saharan Africa recorded the highest neonatal mortality rate, while High-income Asia Pacific had the lowest (Fig. S4). The authors conducted a further analysis of the top ten level-3 causes of neonatal mortality across different regions and found that neonatal disorders were the leading cause of death in all regions in both 2000 and 2021 (Figure 2A). Lower respiratory infections ranked second in 2000 but fell to third by 2021, particularly in Asia, Oceania, and Africa. Congenital birth defects were more prominent in high-income regions (e.g., Asia Pacific, America, Europe), whereas infectious diseases like diarrheal, malaria, meningitis, and tetanus were more frequent in low-SDI regions. While most regions saw declines in cause-specific mortality, several reported significant increases in specific causes between 2000 and 2021 (Figure 2B). These included sexually transmitted infections excluding HIV and diarrheal diseases in Australasia and Western Europe, other unspecified infectious diseases in Central Asia, and congenital birth defects in Southern Sub-Saharan Africa.

### National trends

**Fig. 3:**
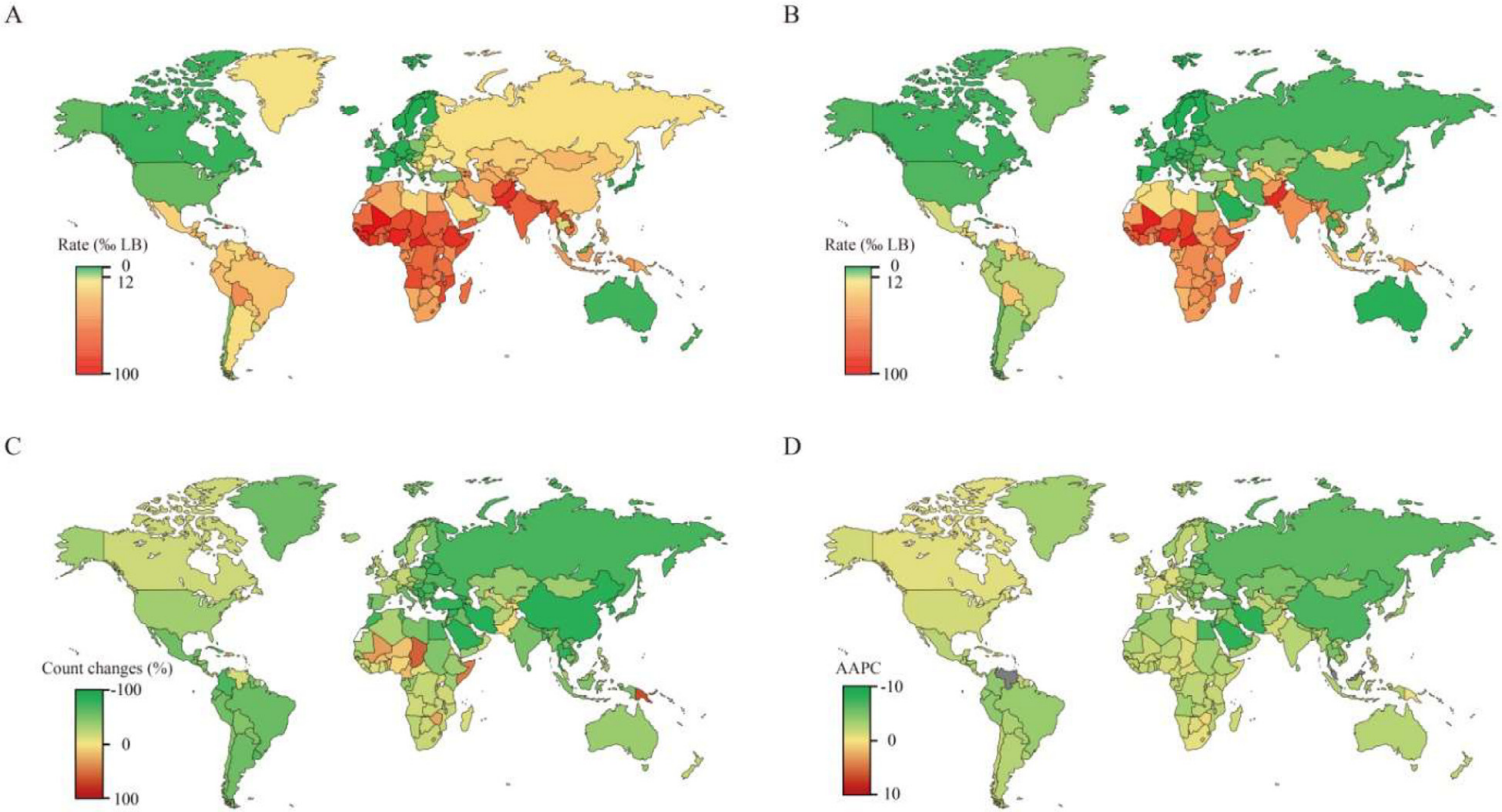
Current status and temporal trends of neonatal mortality across 204 countries and territories. A. The mortality rate in 2000; B. the mortality rate in 2021; C absolute changes in counts; D. the AAPC of mortality rate from 2000 to 2021. AAPC, average annual percentage change; LB, live births.

Neonatal mortality rates exhibited substantial global inequality in both 2000 (range: 2.11–73.60‰ LB) and 2021 (range: 0.61–55.35‰ LB), with the highest rates consistently concentrated in low-income African nations (Figure 3A, B). By 2021, 120 countries (58.8 %) had achieved the SDG 3.2 target. Over the study period, the vast majority of countries experienced a decline in either mortality counts (90.2 %) or rates (92.6 %). However, 20 countries saw an increase in death counts — notably Papua New Guinea, Chad, and Tokelau, where counts rose by > 50 %, and five countries (Brunei Darussalam, Dominica, Guam, Niue, and Zimbabwe) recorded rising mortality rates (Figure 3C, D). In Latin America, neonatal mortality rate has declined steadily since 2000, with considerable heterogeneity across the region. In 2021, Brazil, Chile, and Uruguay have achieved the SDG 3.2 target; however, countries like Bolivia, Venezuela, and Paraguay still report rates exceeding 12‰ LB.

From 2000 to 2021, neonatal preterm birth consistently ranked as the leading cause of death in the majority of countries (148 and 155 countries, respectively), followed by neonatal encephalopathy (54 and 46 countries) (Fig. S5). A notable shift occurred in the Republic of Moldova, where neonatal sepsis and other infections became leading cause of death by 2021.

### Correlations between mortality rate, its AAPC, and HDI

**Fig. 4:**
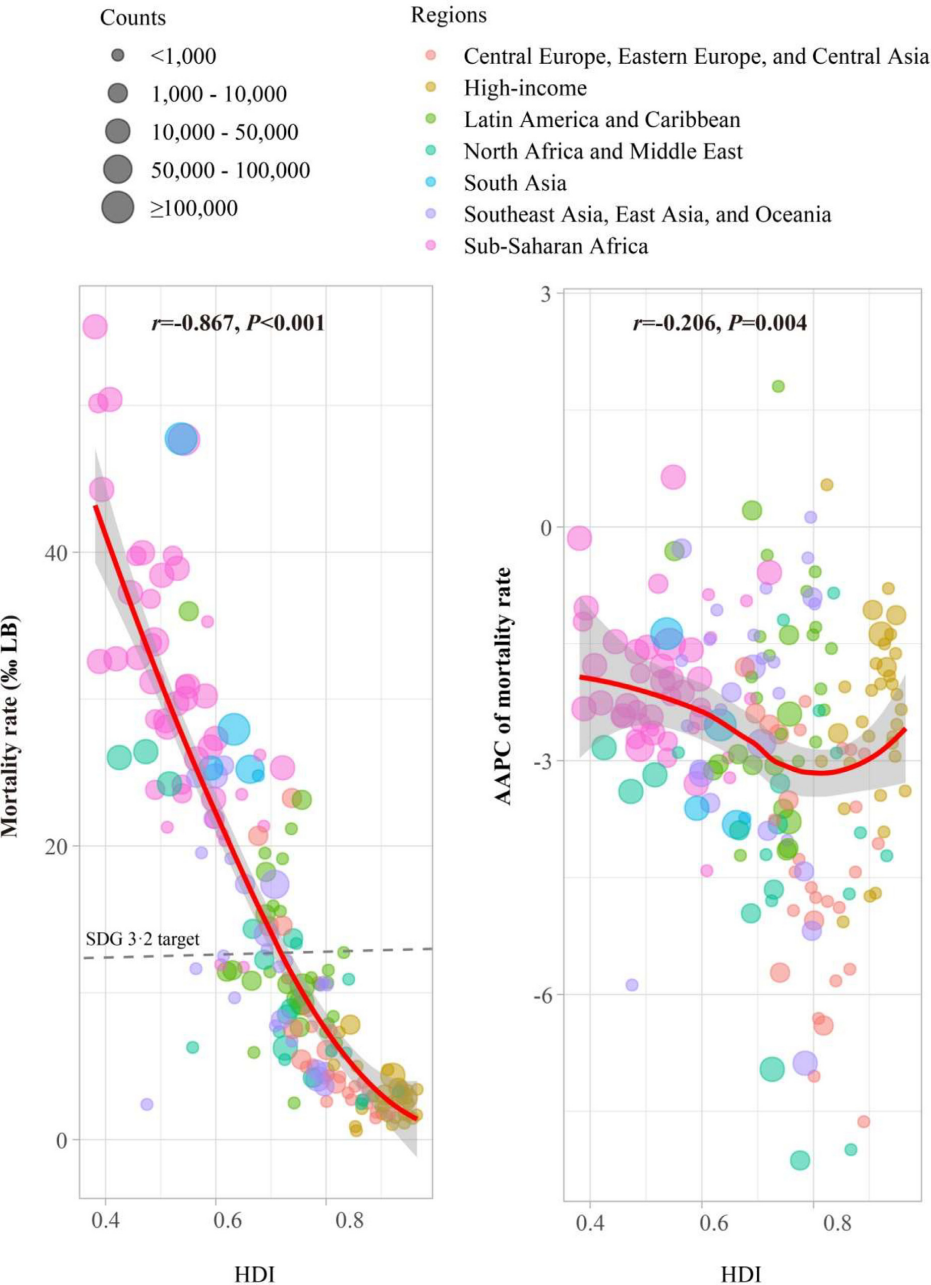
The correlations between mortality rates, their AAPC, and the HDI at the national levels. Pearson's correlation was used to examine the relationships between the mortality rate and the AAPC with the HDI. AAPC, average annual percentage change; HDI, human development index. LB, live births.

As illustrated in Figure 4, the analysis revealed strong negative correlations between mortality rates and HDI in 2021 (*r* = −0.867, *P* < 0.001). Most high-income countries have already achieved the SDG 3.2 target, while countries in Sub-Saharan Africa are still far from reaching this goal. However, the correlation between the AAPC and HDI was significantly weaker (*r* = −0.206, *P* = 0.004).

## Discussion

In order to achieve the SDG 3.2 target, this study provided a comprehensive, multi-level analysis of all-cause and cause-specific neonatal mortality from 2000 to 2021. These findings reveal persistent and profound disparities: by 2021, only half of all countries had achieved the target neonatal mortality rate (≤12‰ LB), underscoring the urgent need for redoubled global effort. The analysis generates a detailed, evidence-based overview of the challenges by integrating cause-specific and HDI data across 204 countries. It also helps pinpoint where progress has changed by identifying key inflection points in the mortality trend around 2011 and 2017. These insights are vital for policymakers and health organizations, clarifying that overcoming the stagnation in neonatal survival requires tailored, dual-track strategies that address both the immediate biomedical causes of death and the underlying social determinants of health.

The global all-cause neonatal mortality rate has declined by an average of 2.1 % per year since 2000, with all regions reporting a decrease. Notably, while 92.6 % of countries experienced a reduction in mortality rates, nine countries maintained stable rates, and five countries saw an increase. This pattern of uneven progress is exemplified in Latin America as well. Despite a steady regional decline since 2000, a persistent gap remains compared to Europe and other high-income regions, alongside profound heterogeneity within the continent. By 2021, countries such as Brazil, Chile, and Uruguay had achieved the SDG 3.2 target, while others like Bolivia, Venezuela, and Paraguay still reported rates exceeding 12‰ LB. In this context of a concurrent global decline in fertility rates [23], ensuring the survival of every newborn becomes even more critical, underscoring the need to sustain and accelerate efforts to reduce neonatal mortality.

Although most regions experienced a broad decline in cause-specific neonatal mortality from 2000 to 2021, several high-income regions reported counterintuitive increases in specific infectious causes. For instance, sexually transmitted infections excluding HIV rose significantly in Australasia and Western Europe, and diarrheal diseases increased in Australasia, Western Europe, and Central Europe. In fact, sexually transmitted infections continue to be the most frequently reported condition to the Australian National Notifiable Diseases Surveillance System, and the numbers continue to rise [24]. The observed increase in mortality from specific infections in several high-income regions is notable and warrants consideration. One possible contributor is the growing challenge of antimicrobial resistance, which can complicate treatment and worsen outcomes for neonatal infections [13]. Additionally, improvements in disease surveillance and diagnostic capabilities over the study period may have led to more complete identification and reporting of cases that were previously undetected. Shifts in maternal demographic and health profiles — such as increasing maternal age and rising prevalence of chronic conditions — may also influence neonatal susceptibility to infection [25].

Neonatal disorders, particularly preterm birth and birth asphyxia, remained the leading causes of death globally in 2021, consistent with prior estimates [1]. However, in the Republic of Moldova, neonatal sepsis and other infections emerged as the primary causes. Although a decreasing trend in the mortality rate of neonatal sepsis and other infections was observed globally from 1990 to 2019, an increasing trend in incidence was noted among neonates [26]. These findings highlight the need to strengthen infection prevention, antimicrobial stewardship, and access to essential treatments, especially in regions where infectious causes remain predominant.

The joinpoint regression identified 2011 and 2017 as significant inflection points in the global decline of neonatal mortality. This nonlinear trajectory suggests that the drivers of progress may have shifted around these periods. The initial steeper decline (pre-2011) likely reflects broad successes in scaling up fundamental interventions, such as skilled birth attendance, neonatal resuscitation, and immunization against major infectious diseases [14]. The slowdown or change in trend thereafter may indicate that further gains are increasingly dependent on tackling more complex and resource-intensive causes, such as preterm birth complications and congenital anomalies, which are now leading causes of death and have seen slower progress [1]. This underscores that sustaining progress requires a strategic evolution from broad, foundational programs to more specialized, integrated care systems capable of addressing the residual burden of disease [27].

The present findings reinforce the critical link between socioeconomic development and neonatal survival, as evidenced by the strong correlation with HDI [28,29]. This association highlights a fundamental inequity in the capacity to tackle major causes of death. For instance, managing complex conditions like congenital anomalies depends on advanced medical resources more accessible in high-HDI settings [30], while preventing deaths from infections or birth asphyxia through basic interventions relies on robust primary healthcare and community systems — core components of human development itself [31]. Thus, elevating HDI is not a separate endeavor but a prerequisite that enables the effective implementation of cause-specific interventions across all settings.

Several limitations should be considered when interpreting the present findings. First, the present analysis is based on modeled estimates from the GBD 2021 study. Although GBD employs rigorous methods to integrate diverse data, the accuracy of estimates in regions with limited vital registration depends on modeling assumptions, as indicated by the wide UI in these areas. Second, the ecological design of the present study limits causal inference. The use of population-level data prevents us from examining individual risk factors or directly attributing mortality changes to specific interventions. Third, while the authors report cause-specific mortality, this analysis does not explore the underlying socioeconomic, behavioral, or health-system drivers of these causes, which are essential for designing fundamental interventions. Finally, the present data extends only to 2021 and therefore do not account for the potential effects of the COVID-19 pandemic on neonatal health systems and outcomes.

In conclusion, this study is poised to make a significant contribution to the field of neonatal health by providing a comprehensive analysis of all-cause and cause-specific mortality rates. The results indicate that, although there has been a significant reduction in neonatal mortality since the 21st century, the absolute number of neonatal deaths remained at 2.19 million globally in 2021. By that year, only 58.8 % of countries had achieved the SDG 3.2 goal. These findings emphasize the need for continued efforts to reduce neonatal mortality, particularly in low- and middle-income countries.

## Data availability statement

The data on all-cause and cause-specific neonatal was obtained from GBD 2021 (https://vizhub.healthdata.org/gbd-results/). The data of HDI at the national level were collected from the United Nations Development Programme (https://hdr.undp.org/data-center/human-development-index#/indicies/HDI).

## Ethics approval and consent to participate

The study used publicly available data and did not involve identified human information; therefore, no ethics approval or informed consent was needed.

## Funding

This work was supported by the Senior Medical Talents Program of Chongqing for Young and Middle-aged (No. YXGD202401), and CQMU Program for Youth Innovation in Future Medicine (W0182). The funder has no role in the conceptualization, design, data collection, analysis, decision to publish, or preparation of the manuscript.

## CRediT authorship contribution statement

**Dongqing Gu:** Writing – original draft, Data curation, Methodology, Formal analysis, Funding acquisition. **Xiaofei Zheng:** Writing – original draft, Validation, Methodology, Formal analysis. **Yan Zhou:** Data curation. **Fengjie Tan:** Data curation. **Rui Gui:** Writing – review & editing, Validation, Methodology. **Linna Wei:** Writing – review & editing, Validation, Methodology. **Lubin Liu:** Writing – review & editing, Validation, Project administration.

## Conflicts of interest

The authors declare no conflicts of interest.

## Appendix Supplementary materials

Image, application 1

## Footnotes

Supplementary material associated with this article can be found in the online version at https://doi.org/10.1016/j.jped.2026.101519.
