# Supplementary material for: Neonatal mortality trends in the 21st century: findings from the Global Burden of Disease Study 2021
Source: J Pediatr (Rio J). 2026 Feb 17;102(2):101519. doi: 10.1016/j.jped.2026.101519 (PMC12926632; doi:10.1016/j.jped.2026.101519)
Supplement: Supplementary file 1 [file mmc1.docx]

**JPED-D-25-00423_Supplementary Material**


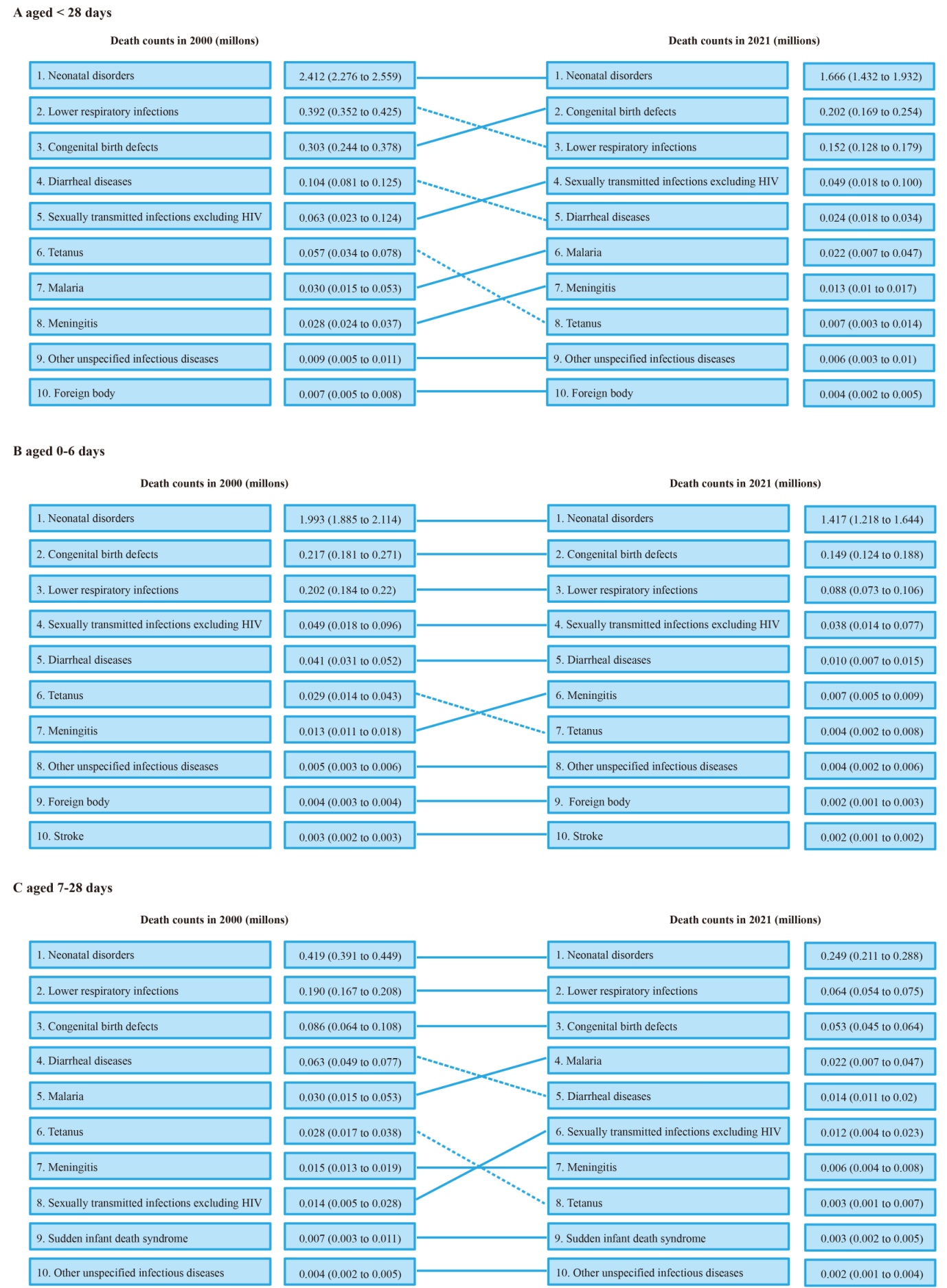


**Figure S1 The top 10 level 3 causes of global neonatal mortality in 2000 and 2021, categorized by age groups:** A. aged < 28 days; B. aged 0-6 days; C. aged 7-28 days.


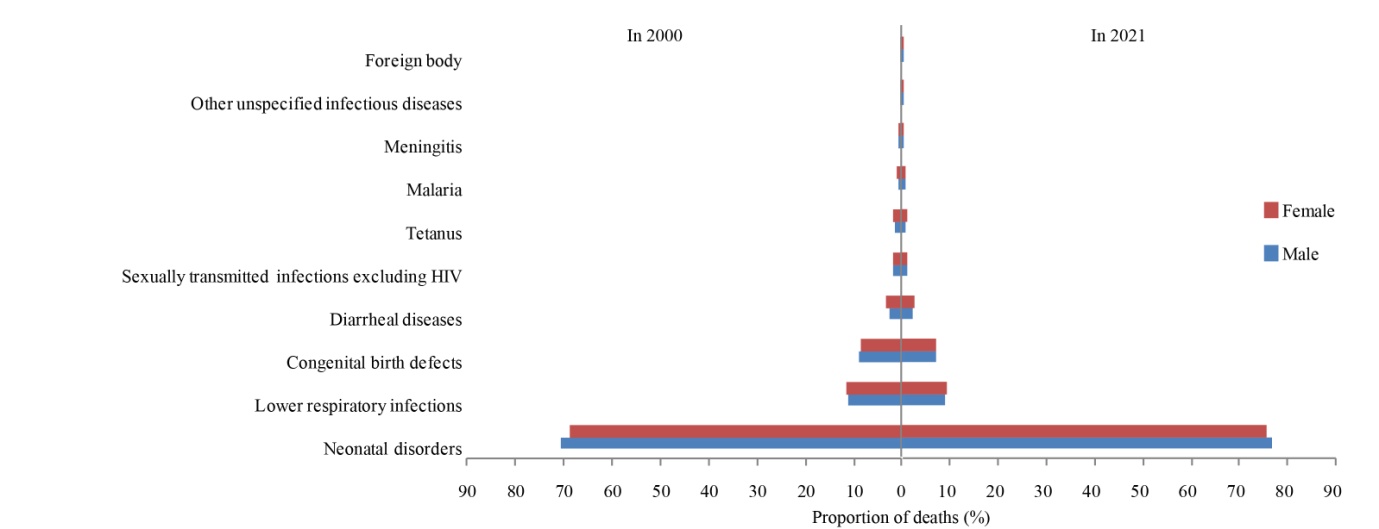


**Figure S2 The top 10 level 3 causes of global neonatal mortality for males and females in 2000, and 2021.**


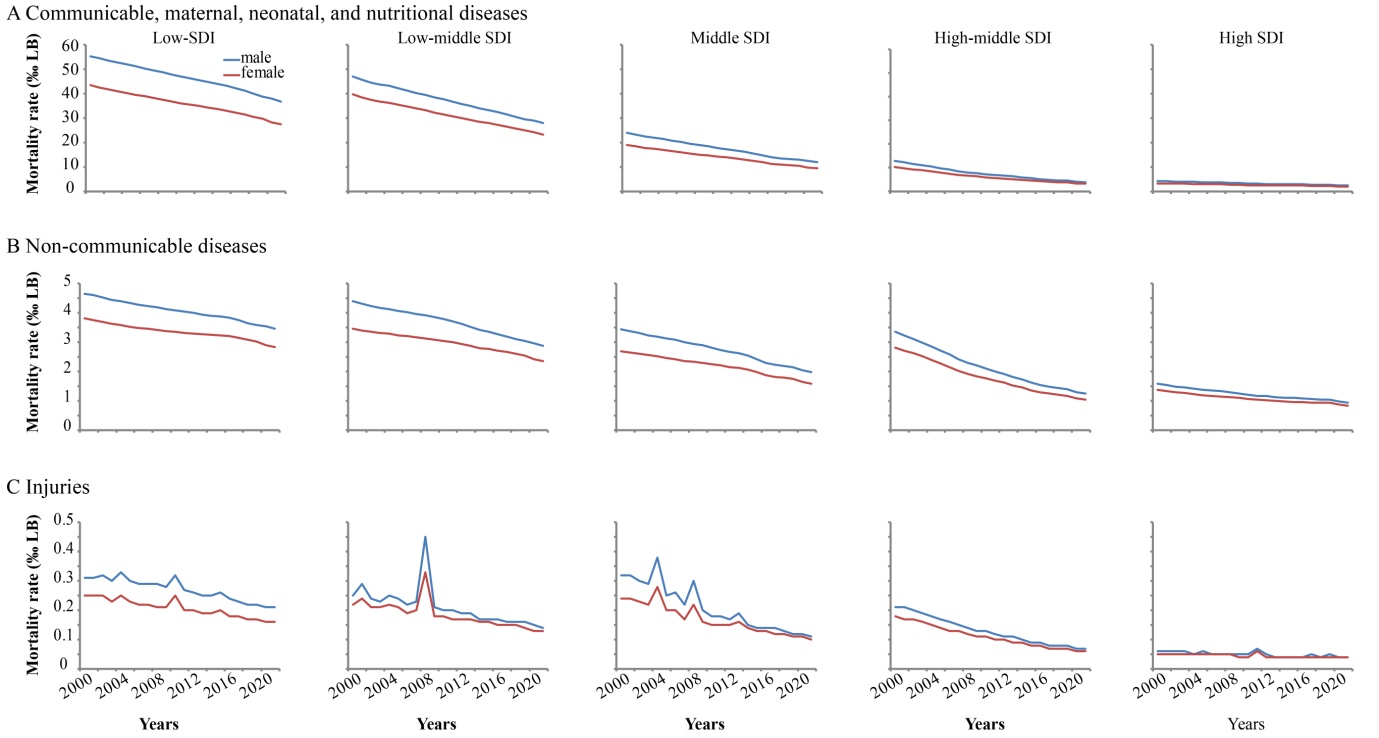


**Figure S3 Level 1 causes of neonatal mortality across different SDI regions.** LB, live births; SDI, Socio-demographic Index.

**
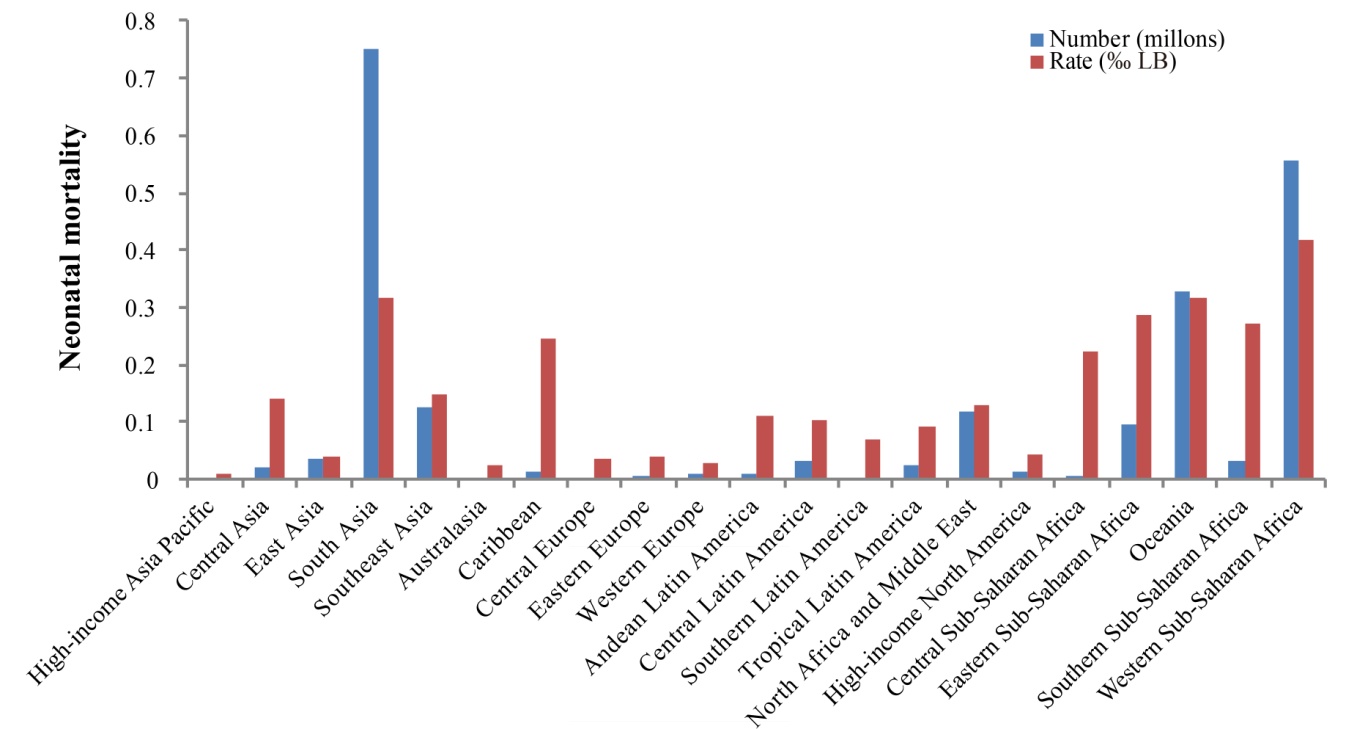
**

**Figure S4 Neonatal mortality by region in 2021.** LB, live births.

**
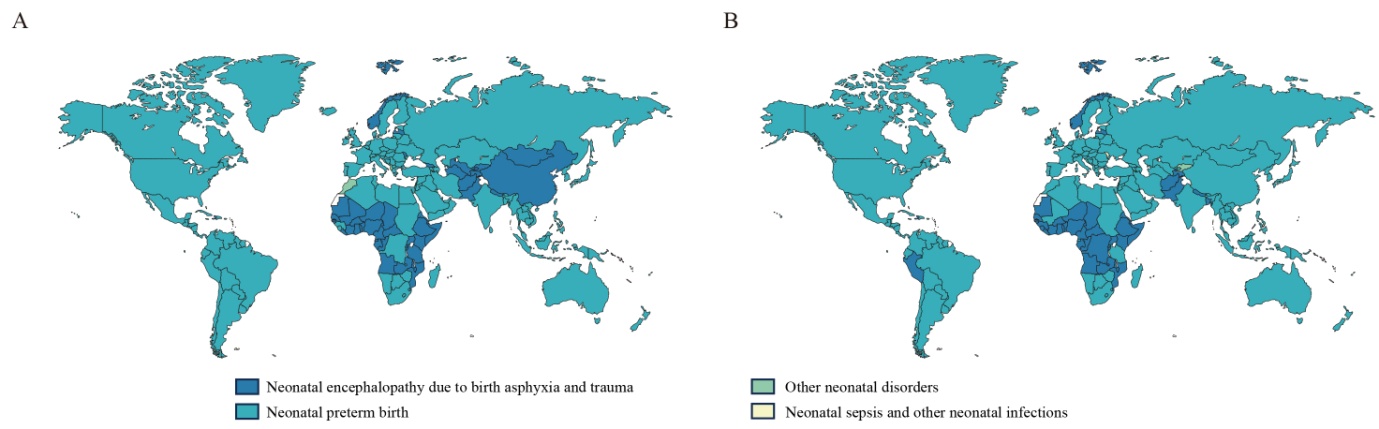
**

**Figure S5 The leading level 4 causes of neonatal mortality in 204 countries and territories in 2000 and 2021. A. The leading cause in 2000; B. the leading cause in 2021.**
